# Optimal COVID-19 lockdown strategies in an age-structured SEIR model of Northern Ireland

**DOI:** 10.1098/rsif.2021.0896

**Published:** 2022-03-09

**Authors:** Gavin M. Abernethy, David H. Glass

**Affiliations:** ^1^ Engineering and Mathematics, Sheffield Hallam University, Sheffield, UK; ^2^ Ulster University, Jordanstown, UK

**Keywords:** compartmental model, epidemiology, lockdown simulation, SARS-CoV-2

## Abstract

An age-structured SEIR model simulates the propagation of COVID-19 in the population of Northern Ireland. It is used to identify optimal timings of short-term lockdowns that enable long-term pandemic exit strategies by clearing the threshold for herd immunity or achieving time for vaccine development with minimal excess deaths.

## 1. Introduction

Epidemiological population models are developed to analyse the characteristics of infectious disease propagation such as the distribution of epidemic sizes [1], to predict the possible course of future epidemics [2], and to determine the efficacy of possible interventions [3]. These are especially useful when there is limited empirical data during the early stages of an outbreak and a rapid response of allocating resources at scale is required to pre-empt the exponential growth of case numbers. In vaccination programmes, mathematical models can indicate what fraction of the population would need to be immunized in order to control the epidemic. In such a way, modelling assists with planning interventions in public health, particularly when trials or direct measures of impact are unavailable [4].

Compartmental population models capture the mechanisms of infectious disease transmission in a population. In such models, compartments represent the different statuses of an individual regarding the disease and so the size of the compartments indicates how much of the population is in a particular state at a given time. Simplest is the SIR (susceptible–infectious–recovered) model, where the infectious compartment accounts for the number of individuals in the population who currently can transmit the disease. A proportion of susceptible individuals who encounter them are infected while infectious individuals recover or perish with a given rate. More sophisticated models of this type may include an ‘exposed’ compartment and may segregate the population by age, gender, or physical location. Such models have been widely used to simulate COVID-19 [5], incorporating complications such as age- or space-structure and multiple infectious compartments to delineate the severity of symptoms. Examples simulating the outbreak in Wuhan include an age-structured SEIIR model [6], and an SEIR model [7] that distinguishes between ‘true’ cases and the estimated fraction of these that are detected. Some approaches separate symptomatic and asymptomatic infections, such as a model calibrated to US states [8]. An age-structured SEIIIR model for Belgium separates the infection into three levels of severity [9], while an SEIRS model allowing for re-infection has been trained on data from Northern Ireland and South Korea [10]. Others are used to quantify the effect of national lockdowns, comparing their impact across countries [11], and testing optimal lockdown and control measures to identify a viable pandemic ‘exit strategy’ [12]. Space-structured network models divide states or nations into multiple smaller communities, with examples including an age-structured model for Scotland [13]; for the UK [14]; for Italy [15]; and for Georgia, USA [16]. Such a spatially structured approach can incorporate non-uniform spatial factors such as age distribution and population density, allowing researchers to forecast the regions of a country likely to be worst affected or to simulate varying regional interventions. An example is a model for the UK [17] that compares the use of national lockdowns with localized lockdowns triggered by regional levels of intensive care unit (ICU) bed capacity.

Mathematical models are employed practically to test the effectiveness of proposed interventions such as further lockdowns, mask use, and social distancing on the possible course of the pandemic. Researchers have sought to quantify the effect of timing and duration of lockdowns [18], finding that timing restrictions within an optimal opportunity window may be critical in controlling peak infections. Others have found that either a longer eradication strategy or a shorter curve-flattening strategy may be preferable when accounting for economic concerns [19]. Throughout 2020, the UK government’s decisions were influenced by modelling by the group at Imperial College London [20], and in November 2020 model projections were the reported rationale for the four-week national lockdown in England. Concerns about a possible second wave, combined with the hope of an imminent vaccine led to further high-intensity ‘circuit break’ lockdowns. As these need to be maximally effective and as short as possible to minimize further economic disruption, a particular application of compartmental models was to identify optimal use of circuit breaks [21,22], while other work has explored switching strategies between lockdowns and keeping communities open [23]. Further research has concerned the question of ‘unlocking’, with both agent-based [24] and SEIR compartmental models [25] favouring exit strategies that gradually release restrictions.

In this paper, we calibrate an age-structured SEIIR model to the age distribution of the population of Northern Ireland and simulate the COVID-19 pandemic throughout 2020 and early 2021, computationally fitting parameters such as infection rates. This model is employed to explore hypothetical implementations of lockdowns. In §4, this involves testing the influence of the duration and intensity of a lockdown and the possibility of restrictions only applying to the more vulnerable members of society. In §5, we test trigger mechanisms that could be used during a future pandemic for implementing restrictions according to the number of hospital occupants. In such cases, we study the impact of the sensitivity of the trigger and the delay until lockdown begins on clinical outcomes such as the overall cumulative deaths from the pandemic.

## 2. Model description

We divide the population of Northern Ireland (approx. 1 894 000) into five 20-year age classes *i* = 1, …, 5, using demographic data [26]. The total population of each age class is considered invariant over the time-frame of the simulations, but they are each subdivided into eight compartments that reflect the status of individuals with respect to the disease, and so the sizes of these compartments are updated according to the epidemiological dynamics described below.

For age class *i*, the population *P*_*i*_ consists of

— *S*_*i*_ susceptible individuals who have never contracted the disease.
— *E*_*i*_ exposed individuals, who are currently infected with the disease but are not yet able to transmit it.
— *I*_*S*,*i*_ subclinical infectious individuals, who have the disease currently and may infect others but who do not display recognizable symptoms. This includes both pre-symptomatic individuals who will later develop symptoms, and asymptomatic individuals, who will never display symptoms.
— *I*_*C*,*i*_ clinical infectious individuals, who have the disease currently, manifest symptoms and may infect others, but who do not yet require hospitalization treatment.
— *H*_1,*i*_ clinical infectious individuals who have been progressed to hospital.
— *H*_2,*i*_ clinical infectious individuals who have been progressed from general hospital wards to ICU.
— *R*_*i*_ recovered individuals who are no longer capable of either spreading the disease or of contracting it.
— *D*_*i*_ deceased individuals.

Thus,

$$P_{i}=S_{i}+E_{i}+I_{S,i}+I_{C,i}+H_{1,i}+H_{2,i}+R_{i}+D_{i}$$

and the total population of the country is given by

$$\$P=\sum_{i=1}^{5}P_{i}\$.$$

Infectious individuals come into contact with (any) individuals of an age distribution governed by a contact matrix *c* [27], and the base probability of infection is determined by the transmission rate *β*. Data for the number of contacts made between individuals of each age class are obtained from an empirical study [27] which estimated contacts separately for ‘home’, ‘work’, ‘school’ and ‘other’ environments for 5-year age classes up to age 80. For this study, we condensed the average of the matrices for the UK and Ireland, with the process fully described in S1.2 of the electronic supplementary material. The final contact matrix *c* and *β* may vary during the simulation, for example as school closures reduce the contact for the youngest age class, and as social distancing reduces the value of *β* across the entire population. Infectious individuals are divided into two categories: subclinical and clinical, who have a relative infectiousness *i*_*S*_ = 1 and *i*_*C*_ = 0.5869, respectively, so that on average 56.1% of infections occur during the pre-symptomatic stage of a clinical case [28–30], given that each such case spends on average 3 days in the subclinical compartment followed by 4 days in the clinical compartment.^1^ Additional infections occur due to subclinical cases that do not progress and may have been undetected in these studies, and due to the emphasis on symptomatic individuals self-isolating a higher transmission rate for the subclinical compartment is not unreasonable. The number of individuals of age class *i* who move to the exposed compartment is further influenced by their susceptibility *α*_*i*_ [31]. Thus, *βα*_*i*_*c*_*j*,*i*_(*i*_*S*_*I*_*S*,*j*_ + *i*_*C*_*I*_*C*,*j*_) would be the rate of new infections generated in class *i* from class *j*, assuming that all members of *i* are susceptible. Summing over the infectious compartments of all classes, and scaling by the probability $S_{i}P_{i}^{-1}$ of an individual of class *i* being susceptible gives the total rate of new exposed cases for this age class.^2^

Exposed individuals become subclinical infectious at rate *σ* (1/5.1 [32,33]). They do not show symptoms at this stage but can transmit the virus to others. With rate *γ*_*S*_ (1/3 [6]) they leave this class, and an age-dependent [31] fraction *ε*_*i*_ develop clinical symptoms, while the remainder join the recovered compartment. Those who have moved to the clinical infectious group then leave it with rate *γ*_*C*_ (1/4 [6]). Of them, an age-dependent fraction *h*_1,*i*_ subsequently require hospitalization, and a fraction *h*_2,*i*_ of that compartment will progress further to ICU [34]. We assume that transmission from hospitalized individuals is negligible as they are isolated. Those in hospital and those in ICU have an age-dependent probability *d*_1,*i*_ and *d*_2,*i*_, respectively, of death [34]. These are recorded as they exit that compartment, after an average of 11 days in general ward and 8 days in ICU (thus leaving with rates *δ*_1_ = 1/11 and *δ*_2_ = 1/8 respectively), based on a major study in China [35].^3^ Any individuals who do not progress to a more serious stage enter the recovered compartment instead.

Probabilities of requiring more serious treatment and of death (*ε*_*i*_, *h*_1,*i*_, *h*_2,*i*_, *d*_1,*i*_, *d*_2,*i*_) are dependent upon age class *i*, but the time spent in each stage and thus the rates of progression (*σ*, *γ*_*S*_, *γ*_*C*_, *δ*_1_, *δ*_2_) are treated as universal across all ages (although some studies indicate that average duration of hospital stays may increase with age [39]). The age-weighted probabilities of progression to hospital, ICU, and death were obtained from the estimates of the Imperial College COVID-19 Response Team [34] using data from March–May 2020 recorded by the COVID-19 Hospitalization in England Surveillance System (CHESS). We have adapted these estimates for our simpler model—combining the probabilities of death in ICU and step-down after ICU, and modifying the probability of death in general ward such that it is only applied to the fraction of patients who are not progressed to ICU. Parameters fixed using external sources are summarized in table 1.

**Table 1.** Summary: parameters chosen based on external data.

parameter	symbol	value	source
incubation rate	*σ*	1/5.1	[32,33]
recovery rate from subclinical	*γ*_*S*_	1/3	[6,40]
recovery rate from clinical	*γ*_*C*_	1/4 (7 days infectious)	[6,40]
progression rate from general hospital	*δ*_1_	1/11	[35]
progression rate from ICU	*δ*_2_	1/8	[35]
age-dependent susceptibility to infection weighting	*α*_*i*_	—	[31]
age-dependent probability of clinical case	*ε*_*i*_	—	[31]
age-dependent probability of hospitalization	*h*_1,*i*_	—	[34]
age-dependent probability of ICU	*h*_2,*i*_	—	[34]
age-dependent probability of death in hospital ward	*d*_1,*i*_	—	[34]
age-dependent probability of death in ICU	*d*_2,*i*_	—	[34]
contact matrices between age classes	*c*	—	[27]

Hence, the rates of change for the compartments of class *i* are given by the following system of ODEs:

$$\begin{array}{rl} \frac{dS_{i}}{dt} & =-\beta \alpha_{i}S_{i}P_{i}^{-1}\sum_{\;j=1}^{5}c_{\;j,i}(i_{S}I_{S,j}+i_{C}I_{C,j})\\ \frac{dE_{i}}{dt} & =\beta \alpha_{i}S_{i}P_{i}^{-1}\sum_{\;j=1}^{5}c_{\;j,i}(i_{S}I_{S,j}+i_{C}I_{C,j})-\sigma E_{i}\\ \frac{dI_{S,i}}{dt} & =\sigma E_{i}-\gamma_{S}I_{S,i} \end{array}$$

$$\begin{array}{rl} \frac{dI_{C,i}}{dt} & =\epsilon_{i}\gamma_{S}I_{S,i}-\gamma_{C}I_{C,i}\\ \frac{dH_{1,i}}{dt} & =h_{1,i}\gamma_{C}I_{C,i}-\delta_{1}H_{1,i}\\ \frac{dH_{2,i}}{dt} & =h_{2,i}\delta_{1}H_{1,i}-\delta_{2}H_{2,i}\\ \frac{dR_{i}}{dt} & =(1-\epsilon_{i})\gamma_{S}I_{S,i}+(1-h_{1,i})\gamma_{C}I_{C,i}\\ & \;+(1-h_{2,i}-d_{1,i})\delta_{1}H_{1,i}+(1-d_{2,i})\delta_{2}H_{2,i}\\ \mathrm{and}\;\;\frac{dD_{i}}{dt} & =d_{1,i}\delta_{1}H_{1,i}+d_{2,i}\delta_{2}H_{2,i}. \end{array}$$

**Figure 1.**
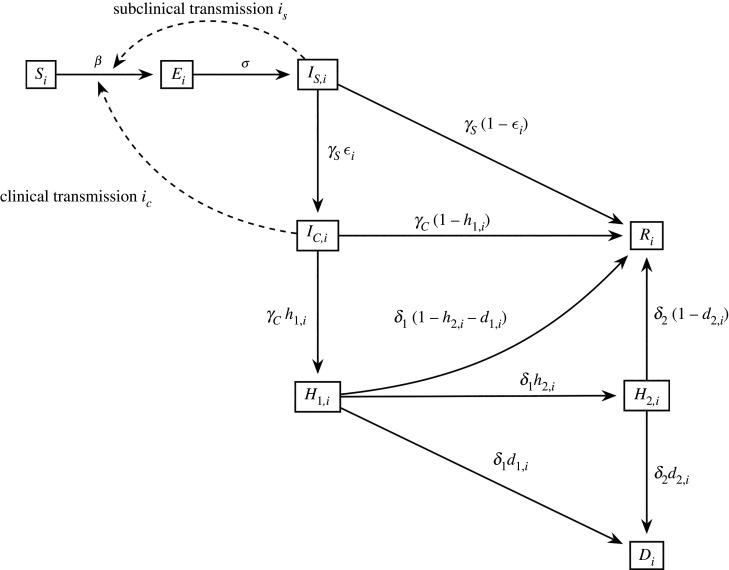
Compartmental flowchart of disease progression within each age class *i*.

These are discretized using the Euler method with step size of one day. The process (excluding transmission between different age classes) is illustrated in figure 1.

For a given simulation, we iterate for 400 days beginning on 1 January 2020. This is divided into nine distinct intervals based on policy in Northern Ireland, and during a simulation the transmission rate *β* must be selected for each of these intervals alongside other parameters that could not be determined *a priori*. These time periods and the parameters to be fitted are described in sections S1.1 and S.1.4 of the electronic supplementary material, respectively.

## 3. Fitted simulation

**Figure 2.**
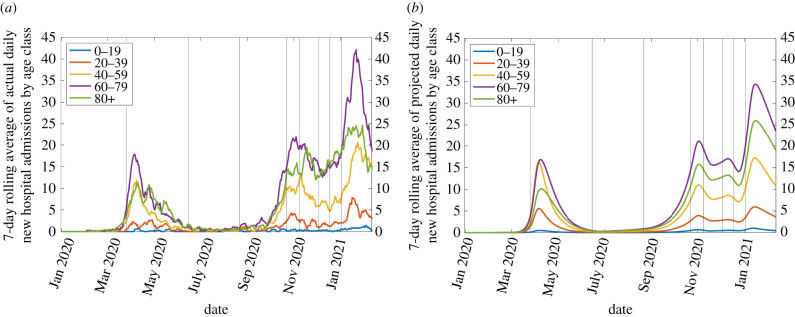
7-day rolling average of daily new hospital admissions. (*a*) Empirical data and (*b*) best fit of the model.

**Figure 3.**
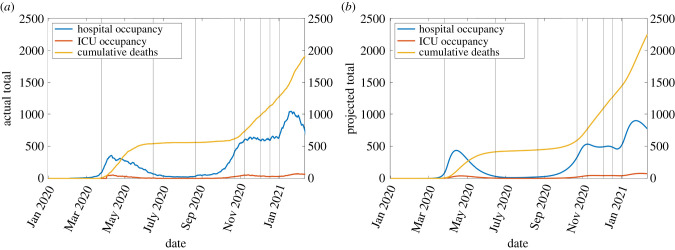
Hospital occupancy, ICU occupancy and cumulative deaths. (*a*) Empirical data and (*b*) projections of the model best fit.

**Figure 4.**
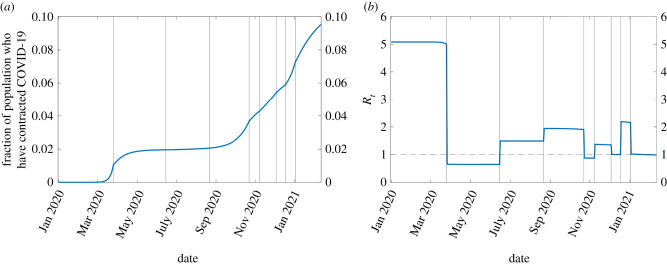
Time series of the spread of the virus according to the best fit of the model. (*a*) Spread of the virus in the population and (*b*) effective reproduction ratio *R*_*t*_.

The simulation which best predicts the 7-day rolling average of hospital admissions (available from the Department of Health [41]) is illustrated in figures 2–4. The accompanying set of parameters are contained in table S3 of the electronic supplementary material, giving a coefficient of determination *R*^2^ = 0.9481. The procedure for identifying these are described in S2.1–2.2 with additional results in S2.3.

Aside from underestimating the number of hospital admissions in those aged 60–80 during early 2021 (figure 2*a*,*b*, purple), the model is able to accurately reproduce the behaviour of the pandemic. The total hospital occupancy during this period is consequently also lower than that recorded (figure 3*a*,*b*, blue). This could also be partly due to longer hospital stays during the winter, noting that such durations vary considerably between countries and studies [36]. Total ICU occupancy and deaths are closely predicted, with only a slight over-estimate of total deaths (figure 3*a*,*b*, yellow) coming from the 60–80 age class (electronic supplementary material, figure S6) while deaths in the 80+ age class are slightly underestimated.

The effective reproduction ratio *R*_*t*_ is calculated daily (figure 4*b*) using the next generation matrix method [42,43]. It is obtained from the largest eigenvalue of a 15 × 15 matrix using the current base transmission rate *β*, the time-dependent contact matrices, and the susceptible proportion of each age class. This yields an estimated *R*_*t*_ of approximately 5.08 during the initial stage of the pandemic, and thus a basic reproduction ratio of *R*_0_ ≈ 5, commensurate with the wide range of estimates in other work.^4^ The measures introduced during the initial spring 2020 lockdown, and the period of strengthened restrictions in late October 2020, successfully reduced the *R*_*t*_ value below one, while the two circuit breaks only just brought it to down to a value of one. Note that *R*_*t*_ is not constant in each time period, decreasing as the proportion of susceptible individuals gradually declines.

**Figure 5.**
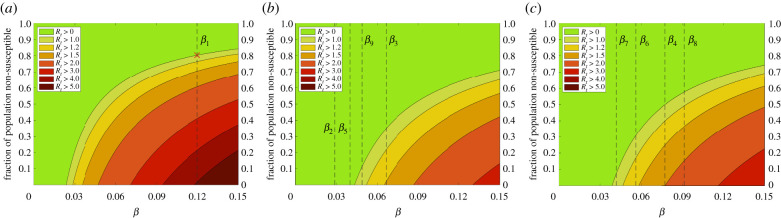
Relationship between *R*_*t*_, transmission rate and the non-susceptible fraction of the population. (*a*) Pre-pandemic social conditions. (*b*) Post-pandemic social conditions, schools closed. (*c*) Post-pandemic social conditions, schools open.

We further calculate the minimum thresholds of the proportion of the population (assuming uniform behaviour across all age classes) who would need to be non-susceptible (whether because of natural immunity, recovery, or vaccination) to control the value of *R*_*t*_ as a function of the transmission rate *β*, and these are presented for each of the three sets of contact matrices used at different periods of the simulation: in figure 5*a* schools are open, and the contact matrices follow their pre-pandemic values. In figure 5*b*,*c*, fitted post-pandemic contacts are used with schools closed and open, respectively. The estimated *β* values at each stage of the pandemic (electronic supplementary material, table S3) are indicated on the appropriate figure panel, so given pre-pandemic rates we would require 80.4% of the population to be immune in order to achieve *R*_*t*_ < 1 (herd immunity) where the virus would fail to establish (figure 5*a*, red cross), while the first lockdown (*β*_2_ line in figure 5*b*) is completely effective and requires no population immunity. This proportion can also be directly estimated by 1 − 1/*R*_0_, for example in [48] who calculated a smaller fraction for the UK based on early estimates of *R*_0_.

Further results of the model are the following.

— 9.5% of the population of Northern Ireland had contracted COVID-19 between the introduction of the virus and early February 2021 (figure 4*a*), whether they had shown symptoms or not. This is in comparison with 104 274 (approx. 5.5% of the population) cases identified by at least one positive laboratory-recorded test by 1 February 2021 [41].
— At no point after 24 March 2020 did either the transmission rate *β* or reproduction ratio *R*_*t*_ return to those of the initial period (figure 4*b*). The most severe spike in cases in winter 2020 was due to a transmission rate approximately 3/4 of that prior to the pandemic, likely due to reduced social interaction. However, at no point were the transmission rate or *R*_*t*_ lower than during the first lockdown, indicating subsequent lockdowns were not as effective in accordance with the perception that they were less severe and the increased transmissibility of subsequent COVID-19 variants.

In electronic supplementary material, S4, the effect of modifying the fixed parameters is investigated. In each case, the fitted values of the infection rate *β* potentially compensate for the effect these changes may have on hospital admissions, but all alternative scenarios yielded a worse fit than the parameters employed in the main body of the paper. Despite this, the time series of total deaths, ICU occupancy, and hospital occupancy remained highly consistent such that in all cases the final number of cumulative deaths by Day 400 lay within 2000–2500, demonstrating that the model’s predicted clinical outcomes are robust to modification.

## 4. Role of intensity and duration of a lockdown

This section investigates how properties impact the effectiveness of a lockdown. During a 1000-day simulation, once the pandemic has been recognized by Day 84 as necessitating a response, we assume a transmission rate *β* = 0.0663 for the remaining days (averaging the fitted rate for non-lockdown post-pandemic periods), except for up to 100 days of lockdown (when schools will be closed). The optimal time for intervention is determined by explicit testing, and the question is how do reductions in total deaths or peak hospitalizations vary with the severity and duration of the intervention? We compare the impact of measures restricted to more vulnerable older age groups with blanket restrictions on the entire population. In each case, the simulation requires the lockdown be activated within 100 days of Day 84, and all simulations last 1000 days so as to avoid merely selecting the circumstances that delay the larger impact of the virus (although even then the virus will not be fully extinguished in most cases).

**Figure 6.**
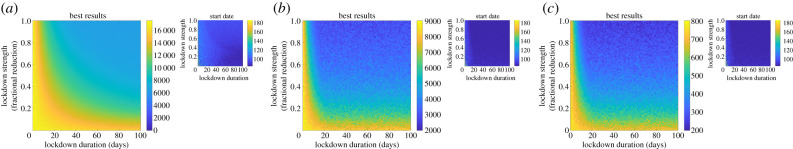
Optimally timed single lockdown in a system with constant inflow. All age classes restricted. (*a*) Total deaths, (*b*) peak hospital inpatients and (*c*) peak ICU occupancy.

**Figure 7.**
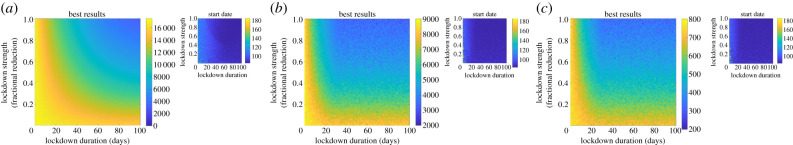
Optimally timed single lockdown in a system with constant inflow. Only the 60–80 and 80+ age classes are restricted. (*a*) Total deaths, (*b*) peak hospital inpatients and (*c*) peak ICU occupancy.

The scenarios illustrated in figures 6 and 7 feature a ‘constant inflow’ of new cases to allow for subsequent re-infections entering from Great Britain and the Republic of Ireland. From Day 31 of the simulation, one additional member is added to a randomly selected age class of the exposed compartment. Testing indicates that the random age can alter projected long-term deaths by up to 1500 by Day 2000; however, the figures are representative of the overall trends. The main figure illustrates the minimized value of cumulative deaths or peak numbers in hospital or ICU for a given lockdown duration (in days, from 1 to 100) and lockdown intensity (defined as the percentage reduction of the transmission rate *β* from its base value 0.0663 during the lockdown, testing from 1 to 100%). The corresponding point in the inset figure indicates the day (between 84 and 183) when the lockdown begins in order to yield this optimal result.

Additional results in electronic supplementary material, S5.1–5.3, include modelling Northern Ireland as a ‘closed system’ with no importation of exposed cases, restricting only the 80+ age class or only the 60–80 age class, and the implementation of a sequence of ten lockdowns each of duration up to ten days.

Unsurprisingly, stronger and longer lockdowns are more effective by every measure across all scenarios. By late March, the strongest measures should usually be implemented as soon as possible (for figures 6 and 7 the dark-coloured top-right of each panel indicates an optimal early intervention). However, when using a single lockdown affecting all ages a slightly later lockdown minimizes overall deaths (inset of figure 6*a*), while the best reduction to peak hospital or ICU occupancy was achieved by intervening as soon as possible (compare with the corresponding inset in figure 6*b*), so the optimal timing of a single lockdown may depend on which outcome is most critical to manage in the short term.

Restricting *only* the contact of all individuals aged 60+ (figure 7) can yield the best reduction of total deaths, but *only* in the case of the strongest and longest-lasting interventions (the top-right corner of figure 7*a* is darker in colour than anywhere in figure 6*a*). This is because it allows spread among the younger population, who then recover, thus building up immunity without incurring a large number of deaths. When the vulnerable groups subsequently exit lockdown, the virus is then less able to spread to them. However, if this strategy is attempted with lockdowns that are insufficient in duration or intensity, the overall effect is actually worse than an equivalent weak–moderate lockdown of all ages. To further demonstrate this, limiting restrictions either to only those aged 60–80 or to only those aged 80+ is less effective than a blanket restriction on the entire population. Thus, targeted restrictions are a risky strategy that may be counter-productive if they are insufficient in strength, duration, or the size of the restricted group. These age-related trends are evidenced for both the closed system and constant inflow models.

## 5. Mechanistic activation of lockdowns

Statistical analysis has indicated that once the virus begins to spread, the precise timing and delays in implementing restrictions can significantly impact case numbers [49], and it is essential to time restrictions based on the true peak incidence [18]. Given possible delays in monitoring and reporting cases [50], we investigate the use of easily trackable clinical outcomes as a mechanistic trigger than could be used to implement restrictions during an ongoing pandemic.

We use the current number of hospital inpatients as the trigger mechanism and study the effect of interventions of varying strengths, the threshold of the trigger that will enable lockdown (between 0 and 2000 hospital inpatients) and the delay between the conditions being met and the beginning of the lockdown (between 0 and 20 days). Low, medium and high interventions reduce the transmission rate by 25% for 15 days, by 50% for 30 days and by 75% for 60 days, respectively. Either a single or an unlimited number of lockdowns are permitted.

Each simulation lasts 2000 days, with a single infection on 30 January 2020, and initial transmission rate *β* = 0.1200. On the 84th day, this reduces to *β* = 0.0663 as in the previous section. This rate reduces further and schools close during a lockdown intervention, and the rate returns (and schools re-open) when the lockdown concludes. No lockdowns are permitted during days 1000–2000 so as to ensure that long-term consequences are accounted for. Caution must be emphasized when interpreting the following results as the model over-estimates deaths, and school holidays are not accounted for. Vaccination is considered in §5.4, but not present in the main set of results.

**Figure 8.**
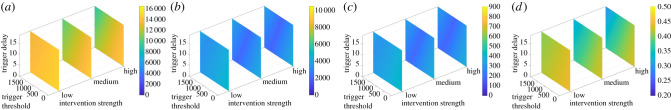
Dynamic lockdowns: closed system–hospital triggers–single lockdown. (*a*) Total deaths, (*b*) peak hospital inpatients, (*c*) peak ICU occupancy and (*d*) disease spread.

**Figure 9.**
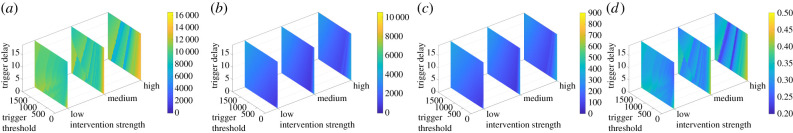
Dynamic lockdowns: closed system–hospital triggers–multiple lockdowns. (*a*) Total deaths, (*b*) peak hospital inpatients, (*c*) peak ICU occupancy and (*d*) disease spread.

Figures 8 and 9 illustrate the effect of lockdowns in a closed system model, using hospital occupancy as the trigger, showing the cumulative deaths, peak hospital and ICU occupancies, and the fraction of the population (disease spread) who have contracted COVID-19 (whether symptomatic or otherwise) by the end of the 2000-day simulation as a function of the trigger sensitivity and the delay between this threshold and the lockdown(s) beginning. Additional outcomes are shown in S6.1 of the electronic supplementary material, along with full results for equivalent scenarios using the number of new daily deaths as the trigger.^5^

**Figure 10.**
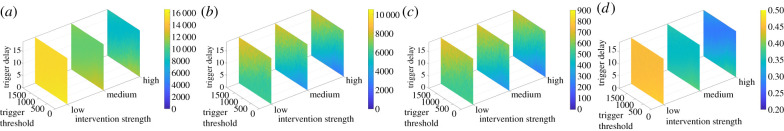
Dynamic lockdowns: constant inflow–hospital triggers–single lockdown. (*a*) Total deaths, (*b*) peak hospital inpatients, (*c*) peak ICU occupancy and (*d*) disease spread.

**Figure 11.**
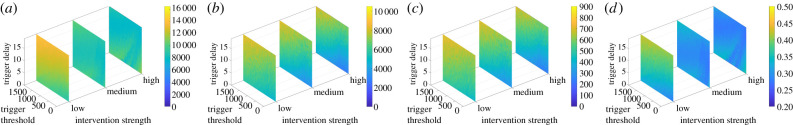
Dynamic lockdowns: constant inflow–hospital triggers–multiple lockdowns. (*a*) Total deaths, (*b*) peak hospital inpatients, (*c*) peak ICU occupancy and (*d*) disease spread.

In figures 10 and 11, we illustrate the equivalent effect of dynamically activated lockdowns in a system where there is a constant inflow of new exposed individuals.

### 5.1. Outcomes of mechanistic lockdown scenarios

Even without eradicating the virus completely, deaths can be reduced over a significant timescale without an indefinite number of lockdowns. With a single lockdown of medium quality (lasting only 30 days), total deaths can be reduced from 16 000 to 10 000 by managing the spread until herd immunity (*R*_*t*_ < 1) is reached with sufficient recovered cases. However, this depends on several years of post-pandemic social distancing, assuming reduced transmission rates rather than a return to pre-pandemic conditions. As it is more realistic to model Northern Ireland as a system which cannot be fully isolated, cumulative deaths will slowly continue to grow, and long-term forecasts are discussed in S6.4 of the electronic supplementary material. Therefore, these investigations primarily concern short-term lockdown strategies to minimize deaths while a permanent solution is developed, such as the vaccination exit strategy separately considered in §5.4.

There is the counterintuitive result that while peak hospital and ICU occupancy is uniformly reduced in a closed system model compared to the equivalent scenario with constant inflow, the propagation of COVID-19 and the accompanying cumulative deaths are *not* always lessened in such a model. In fact, when strong interventions are used, there is greater overall spread of the virus in a closed system and hence greater deaths (compare the strong slices of figure 8*a*,*d* with those of figure 10), while weak interventions revert to the expected patterns. This is because of a surge in the virus that occurs in such a system when strong restrictions are lifted (S6.5 of the electronic supplementary material), which is greatly reduced if there is a constant importation of new cases as these lessen the impact of the lockdown. It is still the case in a closed system that stronger controls are generally more effective than weaker restrictions, but the relative difference is greatly reduced compared to a system with constant inflow.

### 5.2. Deaths, number of lockdowns, and the spread of the virus

Cumulative deaths can be reduced by at least one intervention, best triggered by a high number of hospitalizations (or an intermediate number of daily deaths). Where the thresholds for lockdown are too low, there may be little long-term benefit (see the right-hand edges in figures 8*a* and 9*a*). This is especially the case where there are multiple lockdowns permitted in a closed system. Here, many lockdowns are triggered in succession, covering much of the first 1000 days. If deaths are measured at day 1000, close to the time while measures are in place, this will seem to yield optimal results (S6.6 of the electronic supplementary material). However, once significant time has passed without further restrictions, it is ultimately a poor long-term strategy—observe the many deaths with low-threshold strong restrictions in figure 9*a*. This demonstrates a circumstance where the only gain from lockdown is to purchase time, as these lowest thresholds become optimal again if a vaccination programme is forthcoming (§5.4).

Alternatively, if using high-threshold daily deaths as the trigger the intervention may be too late or even never activated, leading to the worst-case scenario with around 16 500 deaths. This is in agreement with other studies [18] which found that triggering a lockdown within an optimal window of 5–15 days before the peak cases was crucial for significantly reducing peak hospitalizations.

The maximum cumulative deaths across these experiments is approximately 16 500. This is equivalent to the virus spreading to 45% of the population, where there is an *R*_*t*_ value of 0.85 and so even without interventions herd immunity is achieved. This is close to the minimum of 41.6% predicted to achieve *R*_*t*_ < 1 for this value of *β* under such post-pandemic social behaviours in figure 5*c*. The discrepancy is due to non-uniformity in the distribution of cases (and that there will be some overshoot as individuals are infected while the virus is unable to sustain itself), whereby in this worst case proportionally fewer infections are spreading among the vulnerable older age classes according to the contact matrices while the 20–40 age class has the highest proportion of infections. This is also why the upper limit of 16 500 deaths is less than half the maximum possible deaths (electronic supplementary material, S1.3) if the entire population were infected.

### 5.3. Peak hospitalization and intensive care unit occupancy

In a closed system, peak levels of hospital and ICU admission are again minimized not by multiple lockdowns with the lowest trigger threshold as these coincide with a possible resurgence after restrictions are lifted, but instead by multiple lockdowns with a low–intermediate threshold (figure 9*b*,*c*).

In models with constant new cases, high peak hospital and ICU occupancy of around 10 000 and 850, respectively, may be reached if the lockdowns are not activated quickly enough—either because the threshold was too high or if there is too great a delay. As deaths occur later, using these as the trigger requires very low thresholds (electronic supplementary material figure S35*b*,*c*). If hospital admissions are used as the trigger it is more important to ensure that there is low delay in implementation (figure 10*b*,*c*). In each case, the strength of the lockdown is less influential in reducing these peaks than the timeliness of the intervention. This yields two contrasts when managing different outcomes during a pandemic. (i) Intervene earlier while the levels of hospital occupancy are still low to prevent over-burdening the healthcare system,^6^ but hold off until moderately higher thresholds to reduce overall deaths (compare the locations that yield the lowest values in the high-strength slices of figure 9*b*,*c* with that of figure 9*a*). This contrast was also observed in §4. (ii) The strength of an intervention is of greater significance for reducing deaths, while timing plays a greater role in controlling peak occupancies.

### 5.4. Effect of vaccination

**Figure 12.**
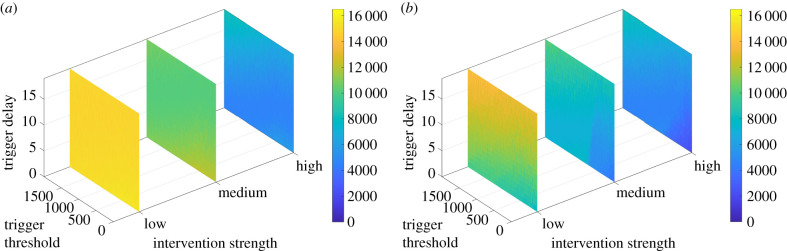
Total deaths using hospital triggers with constant inflow and age-structured vaccination. (*a*) One lockdown and (*b*) multiple lockdowns.

We briefly consider how results are impacted by vaccination, as began in Northern Ireland in December 2020. Every day from 1 January 2021 a constant number *v*(*i*) of individuals from each age class *i* are immediately removed from the pool of susceptible individuals. In the results shown in figure 12, *v* = {200, 400, 650, 1250, 2500} (during January–August 2021 in Northern Ireland, there were 3000–18 600 average daily vaccinations) and we consider the scenario of constant inflow of random age-class infections, using the number of hospital inpatients as the lockdown trigger. Alternative rates of vaccination are tested in electronic supplementary material, S6.7.

Vaccination only has an effect on outcomes if prior lockdowns are sufficient to reduce spread in the preceding period. With a single high-intensity lockdown, vaccination will reduce total deaths from 10 000–12 000 to 5000–6000, of which about 3200 occur during the first year. With multiple lockdowns, it becomes desirable to trigger interventions at the lowest sensitivities and thus to lockdown as strongly as is economically and socially viable to maximally suppress spread until the vaccination exit strategy is available, and the faster the vaccine rollout, the less low that the trigger needs to be in order to prevent additional deaths. Finally, note that there are 3000–4000 total deaths when 1–3 strong lockdowns are triggered by 50–300 hospital inpatients (figure 12*b*), which is not unreasonable given recorded COVID-related deaths in Northern Ireland reached 3000 in January 2022 after 1 year of vaccine distribution.

## 6. Conclusion

We have determined several principles informing the use of lockdowns for controlling future infectious diseases in Northern Ireland, demonstrating significant reductions in total forecast deaths by proper timing of interventions. Furthermore, restrictions targeting only the most vulnerable rather than the entire population may have short-term benefits, provided they are of sufficient intensity and last long enough such that these individuals are protected while the virus spreads among the less vulnerable until they recover. Earlier interventions tend to be more helpful at managing peak demands on healthcare resources, while optimal strategies for lowering cumulative deaths require later initiation of restrictions. Indeed, the exact timing is crucial for controlling the peak number of occupants in hospitals and intensive care, while the greatest factors affecting the number of deaths are more often the duration of the lockdown and how effectively it reduces transmission.

The study is limited in the modelling of vaccination programmes by the restriction of the scope to the short- to medium-term use of lockdowns. Furthermore, an average non-lockdown transmission rate is employed for the duration of the investigation of mechanistically activated interventions, rather than varying with the proliferation of more-transmissible mutations. This limits the interpretation of these results specifically regarding COVID-19 in the UK in 2021–2022, while allowing the principles obtained to be more broadly applicable to future pandemics. Finally, note that the parameter choices tended to predict fewer hospitalizations but greater deaths than observed in Northern Ireland for the 60–80 age group.

Unless and until a vaccination exit strategy is feasible (as for COVID-19), implementing the strongest controls at too low a threshold of the disease’s effect may not be the best route to minimizing long-term deaths—although it may not be apparent in the short term while interventions are still available. Locking down too strong and too early in an isolated society can result in a resurgence when restrictions are lifted if there is a failure to completely eliminate the virus, with potentially higher deaths than if it was permitted to spread to a small extent prior to lockdown. Such hazards observed in a closed system may be applicable to more geographically isolated territories than Northern Ireland.

## Data Availability

The Matlab scripts required to reproduce the primary results can be obtained from the electronic supplementary material [51], which also includes the necessary formatted empirical demographic and epidemiological data sourced from the Department of Health [41] and the Office of National Statistics [26].

## Endnotes

^1^
We have also investigated an alternative where exposed cases become *either* subclinical for 3 days, *or* clinical for 7 days, essentially merging pre-symptomatic cases into the clinical compartment. By tuning *β* a similar age-profile of cases, and overall projections of deaths, hospital and ICU occupants may be recovered. Thus the exact behaviour of the disease in this regard is not critical for the model’s predictive accuracy.

^2^
This formulation is equivalent to $S_{i}^{\prime }=-\beta \alpha_{i}S_{i}\sum_{\;j=1}^{5}c_{i,j}$ $(i_{S}I_{S,j}+i_{C}I_{C,j})P_{\;j}^{-1}$, such as demonstrated in the electronic supplementary material of [27].

^3^
A meta-study [36] found these estimates to vary considerably, with other Chinese studies producing larger estimated lengths of stay, while a smaller number of studies outside China yield lower estimates. Some UK-specific studies suggest average lengths of stay closer to 7–10 for general ward and 10–13 for ICU [37,38] depending on the dataset.

^4^
Early studies in China averaged around 2.2 [44] to 2.6 [45], as did the earliest European studies [46]. However, later modelling indicates an uncontrolled basic *R*_0_ of 4.5 [47].

^5^
Using the current number of ICU occupants yields broadly similar results to using hospital occupants, with a rough conversion of 1000 hospital inpatients as a trigger being equivalent to 75 ICU occupants.

^6^
Not necessarily as low as possible in a closed system, but still definitively lower than the optimal threshold for minimizing deaths. See the location of the darkest colours of figure 8*b*,*c* in comparison to figure 8*a*.
